# Measuring Stress in Health Professionals Over the Phone Using Automatic Speech Analysis During the COVID-19 Pandemic: Observational Pilot Study

**DOI:** 10.2196/24191

**Published:** 2021-04-19

**Authors:** Alexandra König, Kevin Riviere, Nicklas Linz, Hali Lindsay, Julia Elbaum, Roxane Fabre, Alexandre Derreumaux, Philippe Robert

**Affiliations:** 1 Stars Team Institut national de recherche en informatique et en automatique Valbonne France; 2 Département de Santé Publique Centre Hospitalier Universitaire de Nice Université Côte d’Azur Nice France; 3 ki elements Saarbrücken Germany; 4 German Research Center for Artificial Intelligence (DFKI) Saarbrücken Germany; 5 CoBteK (Cognition-Behaviour-Technology) Lab La Fédération de Recherche Interventions en Santé Université Côte d’Azur Nice France

**Keywords:** stress detection, speech, voice analysis, COVID-19, phone monitoring, computer linguistics

## Abstract

**Background:**

During the COVID-19 pandemic, health professionals have been directly confronted with the suffering of patients and their families. By making them main actors in the management of this health crisis, they have been exposed to various psychosocial risks (stress, trauma, fatigue, etc). Paradoxically, stress-related symptoms are often underreported in this vulnerable population but are potentially detectable through passive monitoring of changes in speech behavior.

**Objective:**

This study aims to investigate the use of rapid and remote measures of stress levels in health professionals working during the COVID-19 outbreak. This was done through the analysis of participants’ speech behavior during a short phone call conversation and, in particular, via positive, negative, and neutral storytelling tasks.

**Methods:**

Speech samples from 89 health care professionals were collected over the phone during positive, negative, and neutral storytelling tasks; various voice features were extracted and compared with classical stress measures via standard questionnaires. Additionally, a regression analysis was performed.

**Results:**

Certain speech characteristics correlated with stress levels in both genders; mainly, spectral (ie, formant) features, such as the mel-frequency cepstral coefficient, and prosodic characteristics, such as the fundamental frequency, appeared to be sensitive to stress. Overall, for both male and female participants, using vocal features from the positive tasks for regression yielded the most accurate prediction results of stress scores (mean absolute error 5.31).

**Conclusions:**

Automatic speech analysis could help with early detection of subtle signs of stress in vulnerable populations over the phone. By combining the use of this technology with timely intervention strategies, it could contribute to the prevention of burnout and the development of comorbidities, such as depression or anxiety.

## Introduction

In December 2019 in the Chinese city of Wuhan, a new coronavirus pneumonia, COVID-19, emerged. The pathogen involved is SARS-CoV-2. Here, we will refer to the pathology as COVID-19. COVID-19 has spread very rapidly in China but also in many other countries [1]. On March 11, 2020, the World Health Organization declared that the COVID-19 outbreak had become a pandemic [2].

According to previous studies on SARS or Ebola epidemics, the onset of a sudden and immediately fatal disease could put extraordinary pressure on health care professionals [3]. Increased workloads, physical exhaustion, inadequate personal equipment, nosocomial transmission, and the need to make ethically difficult decisions about rationing care can have dramatic effects on their physical and mental well-being. Their resilience may be further compromised by isolation and loss of social support, risk or loss of friends and relatives, and radical, often worrying changes in working methods. Health care workers are, therefore, particularly vulnerable to mental health problems, including fear, anxiety, depression, and insomnia [4,5]. Initial results estimate that 23% and 22% of health care workers experienced depression and anxiety, respectively, during the COVID-19 pandemic [6].

Paradoxically, health care workers do not tend to seek professional help, and stress-related symptoms are often not immediately reported: “burnout, stress, and anxiety will have to wait.” Most of the time there will not even be a demand for care. Early implicit stress detection is of great importance in this population and would allow for timely intervention strategies in order to prevent escalation and complete occupational burnout.

To measure stress in clinical practice, various scales and questionnaires are available, such as the Perceived Stress Scale (PSS) [7], the Stressful Life Event Questionnaire [8], the Stress Overload Scale [9], and the Trier Inventory for Chronic Stress [10]. However, the present health crisis pushed research teams to investigate the use of new technological tools in this specific population. One possible avenue is the use of automatic speech analysis allowing extraction of voice features during standard consultation or over a simple phone call.

Psychological stress induces multiple effects on the body, including increased muscle tension, increased breathing rate, and changes in salivation rate, which may, in turn, affect vocal production [11,12]. Under psychological stress, voice pitch (ie, the acoustic correlate of fundamental frequency [F0]) usually increases, as it is inversely related to the rate of vocal fold vibration, which stretches under stress and becomes tenser together with an increase in subglottal pressure and vocal intensity [13,14]. Indeed, an increase in voice pitch is the most commonly reported finding in studies examining speech under stress. However, stress can also affect other voice parameters, such as an increase in speech prosody [11,13]. In depression, the analysis of speech characteristics has recently attracted considerable research attention [15-17]. Studies revealed that patients show flattened affect, reduced speech variability, monotonicity in pitch and loudness, increased pause duration, and reduced speech rate [18-20]. A recent study investigated the use of speech parameters extracted from audio recordings to differentiate patients suffering from posttraumatic stress disorder from healthy controls [21].

Thus, the detection of subtle events in the voice may offer a window into assessing the impact of stress in situations where circumstances make it difficult to monitor stress directly but need to be addressed urgently [22].

In this work, we aim to investigate the use of a rapid and remote measure of stress levels in health professionals working during the COVID-19 outbreak, utilizing the automatic analysis of their speech behavior during a short phone call conversation.

Firstly, speech samples of health care professionals were collected over the phone during the COVID-19 pandemic, and various voice features were extracted and compared with classical stress measures. Secondly, based on the extracted features, scores from the completed stress scale that were obtained by participants were predicted. The purpose of this pilot study was to assess whether this technological method could be of interest to support early screening of subtle signs of stress.

## Methods

### Participants

Health care professionals were recruited through outreach telephone calls. They worked during the COVID-19 outbreak in the local university hospital center of Nice, France, in either private practices or as independent workers in the Provence-Alpes-Côte d’Azur region. They could occupy any function in these structures. The only criterion for noninclusion was the subjects’ refusal to participate in the study. Inclusion of participants was carried out from May 5 to June 7, 2020.

The study was approved by the Ethical Board for noninterventional studies of the University Côte d’Azur, France (approval 2020-58). Participants were given all the information about the study prior to the call so they could give informed consent. For those interested, the option for a follow-up call with a clinician was provided.

### Procedure

The telephone calls were made by psychiatrists (n=3) or psychologists (n=1) belonging to the Cognition Behavior Technology research team and the memory clinic of the University Côte d’Azur. Calls lasted about 15 minutes and were composed of the following:

1. An informative part explaining the reasons for the call and its structure and how the study is conducted. The participant’s consent was requested to continue and to proceed with a recording of his or her voice.
2. The Motivation Stress Affect (MSA) questionnaire. The MSA questionnaire is a self-administered questionnaire composed of 11 questions that must be answered by “yes” or “no.” The first five questions assess motivation [23], the next two questions assess depression, and the last four questions assess stress [24].
3. Three open standardized questions: neutral, positive, and negative storytelling. In order to capture natural speech, but within a limited time frame, the participant was asked to talk about something emotionally neutral (ie, describe where he or she is), to talk about a negative event in his or her life, and, finally, to talk about a positive event in his or her life. Each answer should have lasted about 1 minute and was recorded in a secure and encrypted way. It was not specified whether the event had to be experienced during COVID-19; thus, it was open to the participant to recall whatever event first came to mind. These free-speech tasks were used in previous studies [18,25] and allowed for a greater range of induced emotional effects, potentially sensitive to signs of stress and depression. The comparison of speech features between neutral and emotionally loaded questions may give insight into the affective state of participants.
4. The PSS. This scale [7] is a hetero-questionnaire composed of 10 questions to be answered by “never,” “almost never,” “sometimes,” “quite often,” or “often.”
5. An open listening part aimed at exploring certain points in greater depth in order to refine the clinical needs.
6. Decision and advice. Following the above steps, the psychiatrist or psychologist offered or did not offer psychological follow-up depending on whether he or she considered that the patient was at risk of developing or had a mood or anxiety disorder. He or she may also have offered advice on intervention strategies (eg, relaxation, yoga, physical activity, and national call platform for psychological support for caregivers).

### Materials

To perform the phone calls for this study, the phone version of the DELTA application [26] was used. The DELTA solution allows for the use of a dedicated interface in the form of an iOS app to make phone calls and locally record these calls on the internal memory of an iPad. The phone calls were made directly with the iPad and through its internal microphone.

These recordings were then automatically transmitted—the iPad had to be connected to the internet—to the DELTA application programming interface (API) for analysis of acoustic and semantic parameters. Once the analysis was complete, the results were displayed directly on the DELTA interface. The recordings were made locally on the phone, the connection between the interface and the DELTA API was secure and encrypted, and the recordings were destroyed from the DELTA servers once the analysis was complete and the results sent to the experimenter.

### Analysis

Audio features were extracted directly and automatically from the recorded audio signals of the three open standardized questions (see item #3 in the Procedure section). Characteristics were extracted from four main areas:

1. Prosodic characteristics, on long-term variations in perceived stress and speech rhythm. Prosodic features also measure alterations in personal speech style (eg, perceived pitch and speech intonation).
2. Formant characteristics represent the dominant components of the speech spectrum and convey information about the acoustic resonance of the vocal tract and its use. These markers are often indicative of articulatory coordination problems in motor speech control disorders.
3. Source characteristics that are related to the source of voice production, the airflow through the glottal speech production system. These features make operational irregularities in the movement of the vocal fold (eg, voice quality measurements).
4. Temporal characteristics include measures of the proportion of speech (eg, duration of pauses and duration of speech segments), speech segment connectivity, and overall speech rate.

Features were extracted using Python 3.7 (Python Software Foundation) [27] and free and publicly available packages. For the temporal features, the My-Voice Analysis [28] package was used. This package was built off of the speech analysis research tool praat [29]. Temporal features were actualized as the speech rate, syllable count, rate of articulation, speaking duration, total duration, and ratio of speaking to nonspeaking. This package was also used to extract prosodic features, namely the F0 values: mean, standard deviation, minimum, maximum, and upper and lower quartiles. The F0 value is the representation of what is known as the pitch.

Formant features were calculated using the Python Speech Features library [30]. To characterize this aspect of speech, the original sound recording was refit according to a series of transformations commonly used for speech recognition that yield a better representation of the sound called the mel-frequency cepstrum (MFC). From this new representation of the sound form, the first 14 coefficients of the MFC were extracted. The MFC values were extracted given that they describe the spectral shape of the audio file, generally with diminishing returns in terms of how informative they are, which is why we only considered the first 14 coefficients. If we were to select a greater number of MFC values, it would result in a potentially needlessly more complex machine learning model using less informative features.

From each of these waves, the mean, variance, skewness, and kurtosis were calculated for the energy (static coefficient), velocity (first differential), and acceleration (second differential).

The Librosa package [31] was used to calculate the mean, maximum, minimum, and standard deviation of the root mean square value, centroid, bandwidth, flatness, zero-crossing rate, loudness, and flux of the spectrogram, or the visualization of the recording.

The source characteristics were extracted using the Signal_Analysis package, version 0.1.26, to extract the micromovements of the sound wave: harmonics-to-noise ratio (HNR), jitter, shimmer, and glottal pulses. Jitter and shimmer are two features of vocal signals that describe the frequency variation from cycle to cycle of the sound wave and the waveform amplitude, respectively [32,33]. While jitter rises with the growing lack of control of vocal cord vibration, higher shimmer is coupled with increased breathiness. HNR is the ratio between periodic components and nonperiodic components that constitute a voiced speech segment [34]. These components correspond to the vibration from vocal cords and glottal noise, respectively.

Speech features vary naturally between males and females due to differences in the length of the vocal tract. These differences have been leveraged in gender classification through speech analysis based on pitch and formant frequencies [35], HNR [36], linear predictive components, and mel-frequency cepstral coefficients (MFCCs) [37]. Previous work found differences in speech depending on gender in the effects of depression and the effectiveness of classifiers for its detection [38]. This is why this study considers males and females separately.

### Statistical Analysis

The data collected were described using mean and standard deviation for quantitative variables, and frequency and percentage for qualitative variables. Demographic characteristics, such as age and gender, were compared between different groups of caregivers using a chi-square test for qualitative variables (eg, gender) and an analysis of variance performed for quantitative variables (eg, age). Similarly, the data measured for voice and scores were compared between different groups of caregivers. The normality of the collected data was tested using a Shapiro test. In order to test the relationship between the different voice measures and the measured scores, Spearman correlations were used. In addition, to test the link between the voice measures and the therapist’s decision, Student *t* tests or Wilcoxon-Mann-Whitney tests were performed. A *P* value of less than .05 was considered significant. The analyses were performed using the free statistical software RStudio 4.0.0 [39]. Further, regression analyses were performed with the extracted vocal features to determine the error rate for predicting the participants’ stress scores.

## Results

### Participants

In total, 89 French-speaking health professionals, aged between 20 and 74 years, accepted the outreach phone calls and their speech samples were recorded and analyzed. Their demographic characteristics are presented in Table 1.

**Figure 1:**
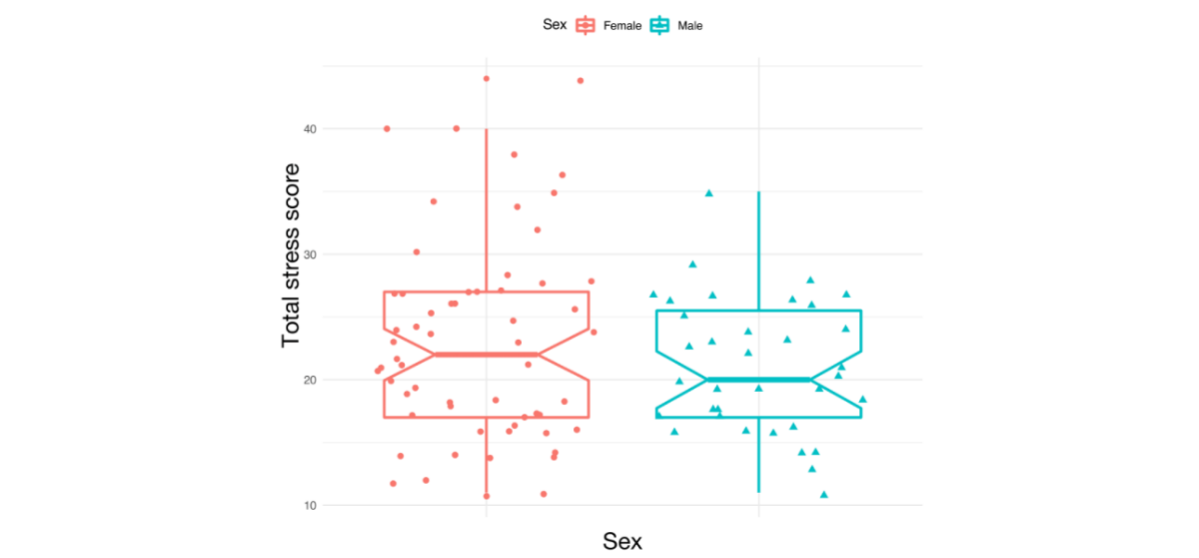
Stress score distribution across genders.

The mean age of the participants was 40.53 years (SD 14.19). The mean stress score on the PSS was 22.43 (SD 7.16) and on the MSA questionnaire was 2.92 (SD 2.09). The majority of the participants scored below 26 on the PSS but above 0 on the MSA questionnaire. Results on the PSS and on the MSA stress scale were proportional. We found that 27% (24/89) of the recorded health professionals experienced intense stress, and 28% (25/89) experienced occasional stress. Only 16% (14/89) of the participants requested a follow-up. The stress level was gender dependent, with females reporting higher stress levels. For males, stress levels tended to drop with age. Figure 1 shows a distribution of the total stress scores across genders. The total stress scores in the female group are more dispersed than in the male group and are generally higher. A total of 14 out of 88 (16%) participants (11/57, 19% of all females; 3/31, 10% of all males) asked for a follow-up call. Their mean PSS score (mean 31.78, SD 7.40) and mean MSA scale score (mean 5.57, SD 1.34) were significantly higher than for those who did not ask for a follow-up, whose mean PSS score was 20.60 (SD 5.63) and mean MSA scale score was 2.38 (SD 1.8).

**Table 1:** Descriptive statistics for participant characteristics (N=89).

Characteristic			Participants, n (%)							*P* value^a^
			Total (N=89)		Male (n=31)		Female (n=58)			
Gender			89 (100)		31 (35)		58 (65)		N/A^b^	
**Education (years) (n=81)**									.03	
	<12	19 (23)		1/28 (4)		18/53 (34)				
	≥12	62 (77)		27/28 (96)		35/53 (66)				
**Timing of call**									.03	
	During lockdown	34 (38)		7 (23)		27 (47)				
	After lockdown	55 (62)		24 (77)		31 (53)				
**Perceived Stress Scale score**									.47	
	Knows how to manage stress (<21)	40 (45)		16 (52)		24 (41)				
	Generally knows how to cope with stress (21-26)	25 (28)		9 (29)		16 (28)				
	Life is a constant threat (>26)	24 (27)		6 (19)		18 (31)				
**Motivation Stress Affect (MSA) scale score**									.99	
	0	23 (26)		8 (26)		15 (26)				
	>0	66 (74)		23 (74)		43 (74)				
**MSA motivation scale score**									.47	
	0	30 (34)		12 (39)		18 (31)				
	>0	59 (66)		19 (61)		40 (69)				
**MSA depression scale score**									.32	
	0	57 (64)		22 (71)		35 (60)				
	>0	32 (36)		9 (29)		23 (40)				
**Follow-up request (n=88)**									.36	
	No	74 (84)		28 (90)		46/57 (81)				
	Yes	14 (16)		3 (10)		11/57 (19)				

^a^Chi-square test or Fisher exact test.

^b^N/A: not applicable; the *P* value was not calculated for gender.

### Correlations

First, vocal and nonvocal features were analyzed in relation to the stress level. The data set was quite small and, therefore, rather than training a classifier, we performed correlation analysis between the features computed for each speech task and the reported stress level. Further, only extracted speech features were considered; a priori, nonmeaningful features, like ID, were removed.

We performed a selection of the top k features based on their descriptive power for the target variable *total stress score*. Vocal features might be gender dependent. Therefore, we performed a selection of top features for male and female data sets separately. We used Spearman correlation, since we had both ordinal and continuous features: the target *total stress score* is ordinal. Since Spearman correlation uses only the ranks of the variables and not their raw values, we could omit the normalization step. We considered absolute values of the correlation coefficient for feature scoring. Results are presented in Table 2.

The main speech parameters correlating with stress levels in both genders were spectral (ie, formant) features, namely the MFCCs. These features characterize the spectrum of speech, which is the frequency distribution of the speech signal at a specific time. MFCCs were derived by computing a spectrum of the log-magnitude mel-spectrum of the audio segment. The lower coefficients represent the vocal tract filter and the higher coefficients represent periodic vocal fold sources [18]. Moreover, in males’ prosodic characteristics, such as the F0, and in females with the positive storytelling, pitch ranges were associated with stress levels.

**Table 2:** Correlation between stress levels and speech features.

Top 10 features for each data set		Task	Spearman correlation
**Female data set**			
	MFCC^a^3 acceleration skewness	Positive story	0.49
	MFCC2 mean	Neutral story	0.44
	Pitch range	Positive story	0.44
	MFCC3 acceleration skewness	Negative story	0.43
	MFCC2 mean	Positive story	0.44
	MFCC5 acceleration kurtosis	Negative story	–0.42
	MFCC2 mean	Negative story	0.43
	MFCC5 velocity kurtosis	Negative story	–0.40
	MFCC3 acceleration skewness	Neutral story	0.39
	MFCC5 velocity kurtosis	Negative story	0.39
**Male data set**			
	Upper quartile F0^b^	Neutral story	–0.54
	Pronunciation posteriori probability score percentage	Positive story	–0.50
	Energy acceleration mean	Positive story	0.52
	Mean F0	Neutral story	–0.51
	MFCC9 kurtosis	Positive story	0.41
	MFCC9 variance	Positive story	–0.44
	Upper quartile F0	Negative story	–0.47
	MFCC4 acceleration mean	Positive story	–0.40
	Upper quartile F0	Positive story	–0.47
	MFCC12 acceleration skewness	Neutral story	–0.42

^a^MFCC: mel-frequency cepstral coefficient; the numbers following MFCC are part of the feature names presenting their location on a spectrum.

^b^F0: fundamental frequency.

For female participants, correlation analyses between negative, positive, and neutral features and the target feature *total stress score* were performed. Among the top 5 features, we have MFCC acceleration skewness, which correlates with the stress level by 0.45 and 0.37 in the positive and neutral tasks, respectively. The other features among top 5 features are task specific. Thus, for each task there is a different set of features associated with stress level.

For male participants, the selection was performed analogously. The top features are task specific as well, and they differ from the features for the female data set. In this sample, we obtained more negatively correlating features than for the female data set; this meant that features, for instance, related to F0 of low value (mean F0 in the neutral story with –0.51, upper quartile F0 in the negative and positive story with –0.47, and upper quartile F0 in the neutral story with –0.54) are associated with high stress scores. In general, low values represent a smaller pitch range.

### Regression

Stress scores were regressed against measurements for positive, neutral, and negative tasks. Similarly, the regression for tasks of different sentiments was performed for groups of female and male participants to allow for possible impacts of gender on stress levels. For the regressors, we used linear, support vector machine (SVM), and random forest regressors to predict the stress scores.

The first regression approximated the stress score by estimating coefficients for each feature in the training data, where greater coefficients indicate a greater influence over the predicted value. Linear regression models are fast, highly interpretable, and commonly used for prediction of stress scores from audio features and speech analysis, according to previous studies [40-42]. The random forest regressor created a number of decision trees that were constructed based on random sampling from the training data; each tree then attempted to determine the best way to predict the scores given the data it received. Each decision tree outputted a predicted value and the mode value was selected. Decision tree methods have shown high accuracy with good interpretability in similar studies where vocal and linguistic features were employed for detection of emotions, social signals, and mental health problems [43-45]. The SVM regressor took each set of features and projected them as a vector onto a space and attempted to find the optimal way to separate the data. The stress score was then based on the distance from that separator. Stress modeling with inputs from physiological sensors or audio sources using SVM has also been previously reported to give high model performance [46-48]. In recent studies, both SVM and random forest provided notably high prediction and classification strength for stress detection using various speech features [49-51].

The caret package from R, version 3.4.2 (The R Foundation), was used for data training and validation. A 10-fold cross-validation was performed and performance was evaluated using the mean absolute error (MAE): the average of the absolute difference between the predicted and actual values from our models for all participants. The score ranges from 0 to infinity, where a score closer to 0 indicates a better-fitting model.

**Figure 2:**
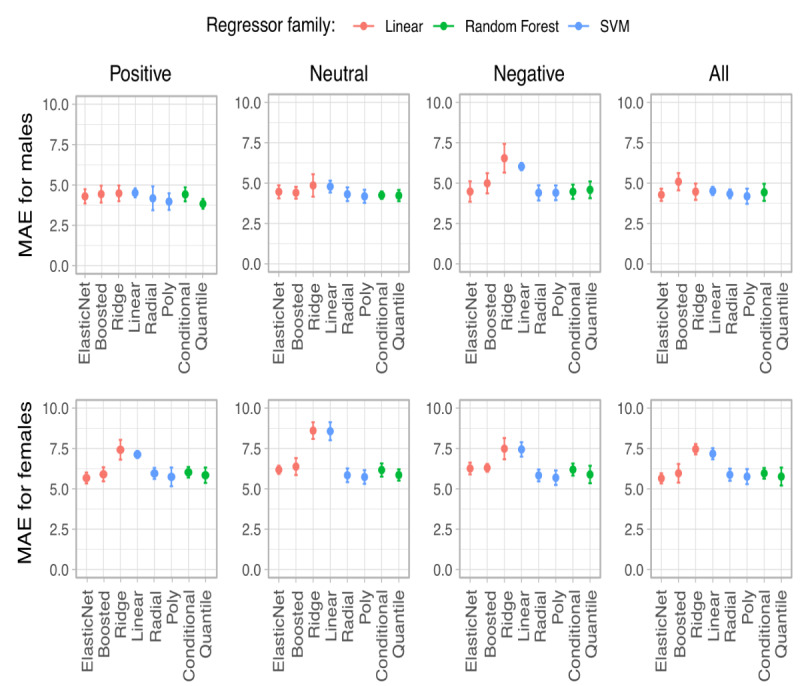
Performances of different computerized regression models in predicting stress levels based on vocal features. Boosted: boosted linear model; ElasticNet: mix of L1 and L2 regularized linear regression; MAE: mean absolute error; Poly: support vector machine with polynomial basis function kernel; Quantile: quantile regression forest; Radial: support vector machine with radial basis function kernel; SVM: support vector machine.

The prediction of total stress scores using all or a subset of tasks among male or female subjects was carried out using various baseline regression models, whose performances were evaluated by the plots in Figure 2, where the MAE values are presented on the y-axis. Overall, the prediction strength in males was better than in females for all sentiments, as shown by a trend of lower errors (lowest MAE for males was 3.84; lowest MAE for females was 5.56). It is notable that stress score regression models based on negative tasks in males and neutral tasks in females performed relatively poorly compared to other tasks. For both male and female participants, using positive tasks for regression yielded equivalent or better results than using all tasks, suggesting that a subset of tasks could be employed for accurate and less time-consuming prediction of stress scores. An overview of the lowest scores for each testing scenario is presented in Table 3.

All regression models outperformed their respective baseline MAE values (4.46 and 6.35 in males and females, respectively). Linear models and the SVM regressor were the most precise for the prediction of total stress scores in general.

**Table 3:** The lowest scores for each testing scenario.

Participant group	Positive tasks		Neutral tasks			Negative tasks		
	MAE^a^ (SD)	Model	MAE (SD)	Model	MAE (SD)		Model	
All	5.31 (0.25)	ElasticNet^b^	5.25 (0.28)	QuantileRF^c^	5.34 (0.35)		PolySVM^d^	
Male	3.84 (0.43)	QuantileRF	4.40 (0.37)	BoostedLM^e^	4.37 (0.43)		PolySVM	
Female	5.56 (0.41)	ElasticNet	5.84 (0.42)	RadialSVM^f^	5.68 (0.45)		PolySVM	

^a^MAE: mean absolute error.

^b^ElasticNet: mix of L1 and L2 regularized linear regression.

^c^QuantileRF: quantile regression forest.

^d^PolySVM: support vector machine with polynomial basis function kernel.

^e^BoostedLM: boosted linear model.

^f^RadialSVM: support vector machine with radial basis function kernel.

## Discussion

### Principal Findings

The purpose of this study was to investigate the potential of using automatic speech analysis for the detection of stress in health care professionals during the current COVID-19 pandemic. This would potentially lead to earlier and timely prevention among this high-risk population. Firstly, speech samples were collected over the phone, and various voice features were extracted and compared with classical stress measures. Secondly, based on the extracted features, scores obtained by participants on the completed stress scale were predicted.

The main outcome of this study was the demonstration of this approach’s feasibility under the given context, as all participants were cooperative and appreciated the initiative of rapidly applying this existing technology to this specific use case. Moreover, from phone call recordings, a number of vocal correlates of stress have been identified, namely in the area of spectral features (ie, MFCC) as well as prosodic features such as F0, which seem to be the most commonly reported features in well-controlled trials [11]. Stress scores could be predicted based on speech features with relatively small errors.

Spectral features characterize the speech spectrum; the frequency distribution of the speech signal at a specific time indicates information in some high-dimensional representation [18]. The features capture information regarding changes in muscle tension and control and have consistently been observed to change with a speaker’s mental state. A few depression studies reported a relative shift in energy with increasing depression severity [52,53].

Another result we obtained was that most identified vocal features were task dependent as well as gender dependent. Interestingly, in the female group, MFCC features seemed to be associated with stress levels during all tasks, meaning that it did not matter what participants were talking about; as long as sufficient speech was captured, meaningful information could be extracted and subtle signs of stress level could be detected. On the other hand, in the male data set, the upper quartile F0 appeared as a task-independent feature sensitive to stress levels. Overall, in the male data set, we observed more features with a negative correlation than we did for the female data set.

Voice production can be divided into three processes: breathing, phonation, and resonance stress [54]. For the second process, phonation, the vocal folds must close and open again to create vibration. The frequency rate of these pulses determines the F0 of the vocal source contributing to the perceived pitch of the sound.

Previous research showed that increased muscle tension tends to be caused by stress [55,56], resulting in a tensing of the vocal folds, which, in turn, most likely causes a raising of F0. A recent review on voice analysis in stress [22] stated that the parameter F0 has been considered as a “universal stress indicator,” whereas increased levels of F0 might be linked with acute bottom-up processes of sympathetic arousal. Similar studies of analysis of phone call recordings during situational stress situations revealed an increase in F0 and intensity with presumed levels of stress [55,57,58]. Our findings seem consistent with the majority of acoustic studies, pointing to F0 as one important marker of stress levels.

However, most correlations we found were with resonance (ie, formant) parameters, which are involved in the quality of sound shaping and vowel and consonant pronunciation and are produced by the muscle activity involved in the shaping of the resonant cavities of the vocal tract system [59]. These parameters are less documented in regard to stress. The MFCC, in particular, can be indicative of breathiness in the voice [60]. Interestingly, one study found a circadian pattern in MFCCs due to sleep deprivation. For this, voice perturbations were compared with classical sleep measures [61] and correlations were found between fatigue scores and MFCCs. This might eventually explain our results, as most participants also reported signs of fatigue during the interviews.

Another study examined speech in students under exam stress and a few days later; in this case, heart rate was measured to control for the actual stress levels. Under stress, students’ heart rates increased, F0 and F0 SD increased, first formant (F1) and second formant (F2) frequencies increased, and MFCCs decreased in relation to baseline levels [62].

It can be hypothesized that given our recorded population who reported relatively mild to moderate levels of stress, rather subtle changes in voice parameters were found and, therefore, weaker correlations were observed. However, it is important to underline that changes in features that we found to be sensitive to stress levels were gender dependent but not necessarily task dependent. They were most likely too small to be detectable by the human ear but were captured by the automatic speech analysis. We assume that by applying this technology to regular check-up calls with people experiencing high stress levels, such as health care professionals, very early signs of stress can be detected in their voices, allowing for timely preventive strategies.

Regression models using vocal features performed relatively well in predicting stress scores, namely in the positive story task for both genders (MAE of 5.31). It shows that the technology could capture indicative patterns from even a short amount of time, possibly even from one task, to recognize tendencies of stress levels in a fragile but healthy population; this represents a promising rapid tool for prediction of stress scores.

### Strengths of This Study

This study is a first step into the early identification of stress in an at-risk population, such as caregivers, who do not directly express their psychological suffering. We can imagine extending this technique to other fragile populations for early screening of stress, such as teenagers who are victims of school harassment or women who are victims of abuse, where timely management could potentially prevent the development of comorbidities, such as depression and anxiety. Moreover, patient populations who have difficulty expressing their problems, such as those with autism spectrum disorder or dementia, could benefit from this technology.

Generally, remote psychological counseling is controversial. Nevertheless, it is becoming necessary due to current economic, social, and health constraints, but has been received by professionals and patients with mixed feelings. Indeed, the nonverbal part of communication is lost and the dynamics of interaction are not the same. However, contrary to these preconceived ideas, we have noticed during this work that it is easier for certain participants to open up and speak about personal issues during these interviews in a liberating manner, similar to a confessional. Not being in the physical presence of the listener may facilitate personal expression, with less fear of being judged. This aspect is very interesting during a screening because it considerably accelerates the process of detection and diagnosing of psychological symptoms.

### Weaknesses of the Study

This project has been rapidly implemented, initially with an approach of qualitative and quantitative data analysis, that should contribute to the early and timely assistance of health professionals during the COVID-19 pandemic. The staff members available to participate in the study were limited. Patient selection was done on a voluntary basis. It is conceivable that the population studied were more concerned about their state of psychological suffering and, therefore, potentially had a selection bias.

Although the voice recordings were made in the middle of the interview without this time being precisely stated, it is possible that some patients may have suspected this, which could have been anxiety provoking and skewed our results. Recording throughout the interview for parameters not affected by the tasks would provide more data and more robust results.

Finally, the obtained correlations can be considered as rather moderate, which makes it difficult to draw any strong conclusions. A larger data set, ideally of a longitudinal nature, with more precise characterization of the speakers is needed in order to verify whether the correlating features represent real markers of stress.

### Future Perspective

For future work, we propose to perform this analysis on a larger data set and to build a prediction model. In case of an insufficient number of observations per stress level, the number of stress levels can be reduced by binning. Binning can also be carried out on characteristic values.

Further studies with acoustic measurements and stress questionnaires at regular time intervals would allow for the analysis of the kinetics of the markers and a better perception of their sensitivity and specificity. In addition, adding clinical measurements of psychiatric symptoms, such as the Diagnostic and Statistical Manual of Mental Disorders, Fifth Edition [63], would make it possible to perceive whether one of the markers is predictive of an anxiety or depression disorder. The use of the tool could be combined with the delivery of preventive strategies, such as physical exercises, adaptation of diet, psychotherapy, meditation, or the use of symptomatic treatments, and it could be employed at the same time for the evaluation of the obtained effects. However, in order to produce a real-world application of this technology, larger validation studies have to be performed to demonstrate clinical meaningfulness by comparing its performance to standardized measurement tools.

## Abbreviations

API: application programming interface
F0: fundamental frequency
F1: first formant
F2: second formant
GSF: Groupe Services France
HNR: harmonics-to-noise ratio
MAE: mean absolute error
MFC: mel-frequency cepstrum
MFCC: mel-frequency cepstral coefficient
MSA: Motivation Stress Affect
PSS: Perceived Stress Scale
SVM: support vector machine
